# Impact of delayed initiation of erythropoietin in critically ill patients

**DOI:** 10.1186/1471-2326-7-1

**Published:** 2007-10-04

**Authors:** Jeremiah J Duby, Brian L Erstad, Jacob Abarca, James M Camamo, Yvonne Huckleberry, Stuart N Bramblett

**Affiliations:** 1 Inpatient Pharmacy Department, Kaiser Permanente, 975 Sereno Dr., Vallejo, CA 94589, USA; 2 The University of Arizona College of Pharmacy, Department of Pharmacy Practice & Science- Pulido, 1295 N. Martin, Tucson, AZ 85721, USA; 3 WellPoint NextRx, 8401 Fallbrook Ave./MS CAAF01-0007, West Hills, CA 91304, USA; 4 University Medical Center, 1501 N. Campbell Avenue, Tucson, AZ, 85724, USA

## Abstract

**Background:**

The purpose of this study was to evaluate the impact of recombinant human erythropoietin (rHuEPO) use for anemia of critical illness at a practice site where delayed initiation is common.

**Methods:**

Retrospective medical record review involving patients treated with rHuEPO for anemia of critical illness. Those patients given rHuEPO or diagnosed with end-stage renal disease (ESRD) prior to ICU admission were excluded. The primary endpoints were rHuEPO use and RBC transfusion patterns.

**Results:**

Complete data were collected for consecutive admissions of 126 patients. Average age (SD) and APACHE II score were 56.5 (18.6) years and 25 (7.8), respectively. The median ICU (IQR) and hospital length of stay (LOS) were 24 (11.25, 39) and 29 (17, 44.75) days, respectively. Treatment with rHuEPO was started an average of 12.5 +/- 10.5 days after ICU admission and given for 3.8 +/- 3.8 doses. Eighty percent of patients were transfused with an average total of 5.42 +/- 5.08 units received. RBC exposure inversely correlated with a lower mean hemoglobin response to rHuEPO. ICU LOS (p < 0.0001), hemoglobin at 24 hours (p = 0.055), transfusion within 48 hours of admit (p < 0.0001), and postoperative status (p = 0.019) were the best predictors of transfusion requirements (r^2 ^= 0.37).

**Conclusion:**

Delayed initiation of rHuEPO for anemia of critical illness resulted in comparable hemoglobin and transfusion benefits. Future studies are needed to establish clinical benefit and role in therapy. RBC exposure may blunt the erythropoietic effects of rHuEPO, potentially frustrating benefits to those of greatest apparent need.

## Background

Anemia of critical illness is a deficiency of blood oxygen-carrying capacity that is clinically characterized by diminished tissue oxygenation and complicated by end-organ dysfunction. The etiology may be categorized into blood loss and reduced red blood cell (RBC) production. Trauma, surgery, hemorrhagic complications, and lab draws compound the effects of functional iron deficiency and blunted erythropoietic response. The incidence and course are obscured by RBC transfusion; however, the average hemoglobin value on admission to the intensive care unit (ICU) is 11 g/dL and tends to approach 10 g/dL at 14 days [1,2].

Allogeneic RBC transfusion is currently one of the principal interventions for acute treatment of anemia of critical illness. Sixty to seventy percent of patients that remain in the ICU beyond 7 days are given RBCs, and critically ill patients receive an average of one to two units per week [1,2]. The severity of anemia and degree of RBC exposure are independently associated with substantially higher risks of morbidity and mortality [1-3].

Recombinant human erythropoietin (rHuEPO) is widely used to promote RBC production and reduce transfusion in the ICU based on a randomized controlled trial (RCT) by Corwin et al. [4]. However, the clinical benefits of rHuEPO remain to be satisfactorily enumerated, prior to establishing cost-effectiveness [5,6]. The actual effects of rHuEPO may differ on the basis that general use commonly deviates from the protocols and patient populations of controlled trials. We observed that rHuEPO use at our institution is frequently delayed and prolonged compared to the largest RCT. The purpose of this study is to evaluate the impact of rHuEPO use for anemia of critical illness at a practice site where delayed initiation is common.

## Methods

This was a retrospective medical record review conducted at a 365-bed academic medical center upon the approval of the IRB. The institution's human subjects committee granted the study exempt status and informed consent was not needed.

### Population

Patients admitted to adult medical or surgical ICUs and treated with rHuEPO for anemia of critical illness between August 1, 2003 and January 15, 2005 were included in the study. Those patients given rHuEPO or diagnosed with end-stage renal disease (ESRD) prior to ICU admission were excluded.

### Design

Potential subjects were identified through a central pharmacy program and ICD-9 codes. Demographic, laboratory, and clinical data for consecutive admissions were collected from the chart and electronic sources, including age, sex, admit diagnoses, medical history, baseline APACHE II and SAPS II scores, rHuEPO and iron administration, RBC transfusion and indication, daily nadir hemoglobin, and ICU length of stay (LOS). The primary endpoints evaluated were rHuEPO use and RBC transfusion patterns during ICU stay.

### Statistical Analysis

From the data collection forms, data was inputted into Microsoft Access for manipulation and compilation of descriptive statistics for the primary endpoint looking at prescribing and transfusion patterns. Intercooled Stata 7.0 (Stata Corporation, College Station, Texas) was used for all other statistical analyses.

Bivariate and multivariate regression analyses were used to evaluate continuous (e.g., age, nadir systolic blood pressure within 24 hours of transfusion, nadir hemoglobin within 24 hours of transfusion, APACHE II score, number of epoetin doses) and dichotomous (e.g., sex, admission to medical or surgical service, iron administration) predictors of transfusion requirements. ANOVA was used to compare hemoglobin concentrations prior to rHuEPO and at two and four weeks following rHuEPO administration. Significance was defined as p < 0.05 for all tests.

## Results

Complete data were collected for 126 patients. Average (SD) age and APACHE II score were 56.5 (18.6) years and 25 (7.8), respectively. Patients were evenly divided between medical and surgical ICUs and were most commonly admitted for respiratory failure, hemodynamic instability, post-operative, and trauma diagnoses. The median (IQR) ICU and hospital length of stay were 24 (11.25, 39) and 29 (17, 44.75) days, respectively.

Treatment with rHuEPO was started an average of 12.5 ± 10.5 days after ICU admission and given for 3.8 ± 3.8 doses (Table 1). An average of 120,000 ± 107,323 units of rHuEPO were administered per patient treatment course at an estimated cost of 1,200 ± 1,073 U.S. dollars. Iron studies were performed on 19.0% of patients and 39.7% of patients concomitantly received iron supplementation.

**Table 1:** Course of Hospital Admission

N = 126	Mean (SD)	Median (IQR)
ICU LOS	28.8 (24.8)	24 (11.25, 39)
Hospital LOS	35.1 (30.3)	29 (17, 44.75)
Nadir hemoglobin (g/dL)	7.71 (0.94)	7.75 (7.3,8.3)
rHuEPO		
Start day (from ICU admit)	12.5 (8.2)	8 (6, 15)
Number of doses	3.8 (3.8)	3 (1, 4)
Days of treatment	23.24 (21.73)	14 (1, 22)

**Figure 1:**
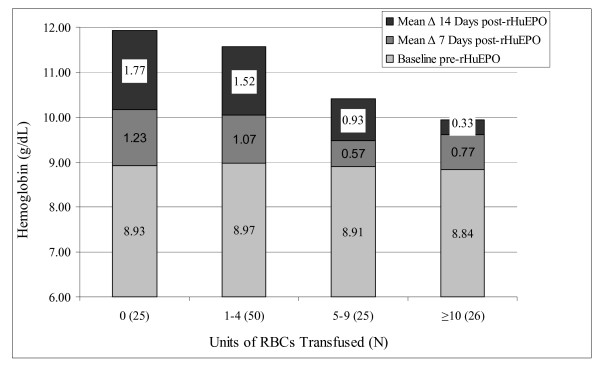
Hemoglobin changes relative to number of units of red blood cells transfused.

Eighty percent of patients were given at least one RBC transfusion in the ICU. Patients received an average of 2.5 ± 2.3 transfusions in the ICU for an average total of 5.42 ± 5.08 units of RBCs or 3.00 ± 3.69 units of RBCs prior to initiation of rHuEPO and 2.42 ± 2.98 units. Transfusion was associated with a diminishing response to rHuEPO such that incrementally higher RBC exposure inversely correlated to a lower mean response in hemoglobin at 7 and 14 days after initiation of rHuEPO (Figure 1).

The mean hemoglobin was 11.06 ± 2.52 on ICU admission, 8.92 ± 0.96 the day of rHuEPO initiation, 10.09 ± 1.42 at 14 days after starting rHuEPO, and 10.08 ± 1.42 at ICU discharge. The mean change in hemoglobin was 1.15 g/dL (p < 0.0001) at 14 days and 1.22 g/dL (p = 0.0003) at 28 days after initiation of rHuEPO.

Four independent variables had statistically significant relationships with transfusion requirements (i.e. units transfused) using simple linear regression: length of ICU stay (p < 0.0001) with an R squared of 0.19, length of hospital stay (p < 0.0001) with an R squared of 0.20, APACHE II score (p = 0.01) with an R squared of 0.05, and the number of epoetin doses administered (p < 0.0001) with an R squared of 0.1. Using multiple linear regression the best model with an adjusted R squared of 0.37 involved the following independent variables as predictors of transfusion requirements: length of ICU stay (p < 0.0001), hemoglobin concentration at 24 hours (p = 0.055), transfusion within 48 hours of admit (p < 0.0001), and postoperative status (p = 0.019).

There was a significant increase in hemoglobin concentrations immediately preceding epoetin administration compared to hemoglobin concentrations at 2 and 4 weeks after epoetin administration (8.9 g/dL, 10.1 g/dL, and 10.4, respectively, p < 0.0001).

## Discussion

In contrast to the largest RCT by Corwin et al, rHuEPO use at our institution appeared to be reserved for patients with a substantially greater severity of illness, ICU LOS, and transfusion burden [4]. The most striking difference in treatment practices was the marked delay in starting rHuEPO such that average initiation was nearly ten days later than that established by the RCT. However, the prolonged ICU LOS experienced by patients in this study resulted in an average of 1.24 additional dosages received compared to the RCT. There was a 30% higher incidence of transfusion and a more than twofold greater average total volume of RBCs given compared to the RCT. The average change in hemoglobin from baseline (pre-rHuEPO) to 28 days (1.44 ± 1.62 g/dL) after starting rHuEPO was similar to that found in the RCT. Further, the transfusion requirements found in the RCT for placebo vs. rHuEPO (3.0 vs. 2.4) were identical to those observed pre- vs. post-rHuEPO (3.0 vs. 2.4). These similarities in transfusion patterns and hemoglobin response are similar despite the substantial disparities in rHuEPO use and severity of illness.

One of the most intriguing findings was the apparent inverse correlation between transfusion and hemoglobin response to rHuEPO. Corwin et al have consistently noted substantially higher hemoglobin levels in patients treated with rHuEPO, despite greater transfusion exposure to placebo groups [4,7,8]. The simplest explanation of this phenomenon is that rHuEPO produces higher hemoglobin levels that result in fewer transfusions. Conversely, transfusion may have a blunting effect on erythropoiesis that depresses hemoglobin levels and leads to additional transfusions, as scattered reports suggest [9-11]. In effect, rHuEPO may help break the cycle of diminishing hemoglobin, consequent transfusion, blunted erythropoiesis, and further transfusion. Consequently, however, those patients that experience the greatest transfusion burden and, therefore, have the greatest appeal for medical intervention may derive the least benefit from rHuEPO. Clearly, the relationship between rHuEPO, erythropoiesis, transfusion, and hemoglobin is extremely complex and warrants further examination based on this and other hypothesis-generating findings.

The principal limitations of our study stem from the retrospective observational design and the lack of a control group; however, concerted efforts were made to reduce potential bias and inaccuracy. First, an emphasis was given to gathering objective data that was prospectively collected (e.g. hemoglobin levels). Second, subjects with complete medical records were enrolled by consecutive admission to minimize selection bias and the possibility of data omission.

## Conclusion

The use of rHuEPO in clinical practice may vastly vary from the model of the RCT. However, delayed initiation of rHuEPO for anemia of critical illness appeared to result in comparable hemoglobin and transfusion benefits. In order to maximize the potential benefit of rHuEPO it is essential to identify patients at greatest risk of a prolonged ICU course and transfusion burden. Further, RBC exposure may blunt the erythropoietic effects of rHuEPO, potentially frustrating benefits to those of greatest apparent need.

## Competing interests

The author(s) declare that they have no competing interests.

## Authors' contributions

JD participated in the design of the study, performed data collection and analysis, and drafted the manuscript. BE conceived of the study, participated in its design and coordination, performed data analysis and drafted the manuscript. JA participated in the design of the study and performed data analysis. JC participated in the design of the study and performed data collection. YH participated in the design of the study and performed data collection. SB participated in the design of the study and performed data collection. All authors read and approved the final manuscript.

## Pre-publication history

The pre-publication history for this paper can be accessed here:


